# Immunomodulatory role of estrogen in ischemic stroke: neuroinflammation and effect of sex

**DOI:** 10.3389/fimmu.2023.1164258

**Published:** 2023-04-25

**Authors:** Xiaojun Zhong, Yulin Sun, Yajun Lu, Lei Xu

**Affiliations:** ^1^ Department of Neurosurgery, Zhejiang Rongjun Hospital, Jiaxing, China; ^2^ Department of Internal Medicine, Sunto Women & Children’s Hospital, Jiaxing, China; ^3^ Department of Neurology, Zhejiang Rongjun Hospital, Jiaxing, China

**Keywords:** ischemic stroke, estrogen, 17β-estradiol, neuroinflammation, neuroprotection, immunomodulation, therapy

## Abstract

Although estrogen is predominantly related to the maintenance of reproductive functioning in females, it mediates various physiological effects in nearly all tissues, especially the central nervous system. Clinical trials have revealed that estrogen, especially 17β-estradiol, can attenuate cerebral damage caused by an ischemic stroke. One mechanism underlying this effect of 17β-estradiol is by modulating the responses of immune cells, indicating its utility as a novel therapeutic strategy for ischemic stroke. The present review summarizes the effect of sex on ischemic stroke progression, the role of estrogen as an immunomodulator in immune reactions, and the potential clinical value of estrogen replacement therapy. The data presented here will help better understand the immunomodulatory function of estrogen and may provide a basis for its novel therapeutic use in ischemic stroke.

## 1 Introduction

Stroke is the leading cause of morbidity and mortality after cardiac diseases and cancers, affecting 15 million individuals worldwide annually (1). As the risk of stroke increases with age, increasing life expectancy and rising aging population will significantly burden the economy (2). Reportedly, the economic burden of stroke has been estimated to reach $184.1 billion by 2030 (3). Moreover, ineffective treatments of stroke along with the aging of the population will increase the risk of mortality and long-term disability in the future (4). Currently, the primary barrier to establishing effective therapeutic strategies is the limited understanding of mechanisms underlying secondary neuronal damage after stroke-induced central nervous system (CNS) injuries (5). In addition, numerous preclinical studies have failed to translate into clinical use owing to the enrollment of only male animals; this emphasized the consideration of sex differences in stroke etiology. Currently, the Stroke Therapy Academic Industry Roundtable guidelines recommend considering age and sex differences when translating preclinical results in clinical trials (6, 7). Indeed, accumulative evidence has revealed that sex is an important factor in the etiology of stroke-induced CNS injuries (8). Therefore, a better understanding of the potential effect of sex can improve clinical prognosis and help establish novel therapeutic strategies. This study provides a comprehensive review of the mechanisms underlying sex-related differences observed in post-stroke CNS injuries, including genetic alteration, estrogen-mediated immune regulation, and downstream signaling pathways. Moreover, the review summarizes the anti-inflammatory effects of estrogen on the brain and emphasizes the clinical potential of estrogen-based therapeutic strategy in patients with stroke.

## 2 Incidence and severity of ischemic stroke based on sex

According to epidemiological data, stroke is a sexually dimorphic disease (9, 10). Although the incidence of stroke is much higher in men, women have been reported to have severe CNS injuries, higher morbidity and mortality, and greater post-stroke neurobehavioral deficits. However, according to the Greater Cincinnati/Northern Kentucky Stroke Study and data from the National Institutes of Neurological Disorders and Stroke, approximately 55,000 more women suffer from stroke annually than men, owing to the longer life expectancy in women (11). Although the incidence of stroke is higher for men, women with stroke have a higher mortality rate (58%), poorer recovery, and greater long-term disability (12). Currently, the incidence rate of stroke has decreased from 19.5% to 14.5% in men, but it has only modestly decreased from 18.0% to 16.1% in women (13). Moreover, women in the age groups of 19–30 and 45–54 years have a higher risk of stroke than men in the same age groups. This may be attributed to the alterations in estrogen status during this age. Particularly, the rapid decreases and alterations in estrogen levels lead to several disorders, which eventually increase the risk of stroke (14).

Although estrogen is a sex hormone in females that regulates reproductive function, it also plays a role in several pathological processes, especially in the CNS (14, 15). Accumulative evidence has demonstrated that estrogen, especially 17β-estradiol, has a protective role in brain injuries after ischemic stroke (16). This protective effect of 17β-estradiol is mediated by several mechanisms, including the regulation of local and systemic immune responses after stroke onset (17).

## 3 Effect of sex on post-stroke outcomes

It has been well documented that the sex chromosome complement is responsible for sexual dimorphism (XX for female and XY for male) (18). Accordingly, a previous study has assessed sex-related differences in the expression of X chromosome genes in patients with ischemic stroke (19). The study found that in females, X chromosome-related genes were enriched in TNFR1, interleukin (IL) 17, and natural killer cell signaling pathways, whereas in males, the X chromosome-related genes were highly expressed in pathways involved in cell development, cellular migration, and pro-inflammatory reactions (20, 21). Moreover, it has been demonstrated that genes associated with the X chromosome are responsible for cellular processes occurring after a stroke, including the activity of NF-κB activating protein, tissue inhibitor of metalloproteinase 1, and IL-1R-associated kinase (22). Specifically, the X chromosome contains several estrogen-response element (ERE) sequences. A previous study has identified 12,515 ERE sites in the human genome and 11,810 in the mouse genome. Yang et al. assessed 300 mice and reported more than 10,000 genes exhibiting sex-dependent expression in somatic tissues (23). Reportedly, approximately 13% of genes in the brain contribute to sexual dimorphism, including 355 in females and 257 in males (24).

To date, several studies have focused on the effects of sex hormones on post-stroke outcomes. Studies have additionally revealed the role of genetic sex differences in post-stroke outcomes. Reportedly, the sex hormone estrogen plays a critical role in sexual dimorphism after vascular attacks; however, this effect of estrogen is not fully accounted for in post-stroke outcomes between males and females. Based on experimental studies, several cell death-associated signaling pathways function independently of sex hormones but have been found to function differently in both sexes. In males, ischemia-induced cell death is predominantly triggered *via* the activation of poly ADP-ribose polymerase (PARP-1), an NAD-dependent DNA repair enzyme. The activation of PARP-1 induces further DNA damage, which results in the release of mitochondrial apoptosis-inducing factor and its subsequent translocation into the nucleus (25). Under the condition of ischemic stroke, PARP-1 activation can be observed in both sexes; however, the neuroprotective effects of this pathway were abrogated in males (26). Moreover, the inhibition or genetic deletion of PARP-1 induced these neuroprotective effects only in males, suggesting a distinct mechanism of the PARP-1 pathway in this sex (27). In addition, the caspase-mediated apoptotic pathway is the predominant mechanism underlying cell death under the conditions of ischemic stroke-induced brain injury.

Cytosolic cytochrome C (Cyto C) is an essential component of the intrinsic caspase pathway. After a stroke, neurons exhibit greater resistance to nitrosative stress and higher Cyto C levels in females than males (28). The main subjects in the study were postmenopausal or old women or ovariectomized (OVX) female models. The lack of estrogen leads to a significant increase in Cyto C (5). Moreover, neuronal responses differ according to sex. A previous study has revealed that after stroke, the caspase expression pattern in females was different from that in males. In addition, pan-caspase inhibitors preferentially protected females, but no such significant effect was observed in males. On the contrary, post-stroke males exposed to oxygen-glucose deprivation were found to be preferentially protected by neuronal NOS (nNOS) inhibitors, whereas females were not (29). Of note, these sexually dimorphic protective effects of caspase inhibitors can be observed in intact females as well as in ovariectomized (OVX) females and females who receive estrogen replacement therapy (ERT), indicating that these neuroprotective effects are independent of sex hormones (30).

## 4 Downstream signaling pathways of estrogen

Estrogen is a lipophilic steroid hormone that can easily diffuse across cellular membranes, especially the blood–brain barrier (31). Naturally, estrogen is synthesized from cholesterol in the ovaries and occurs in three forms: estrone, estradiol, and estriol (32). Estrone is also known as E1, estradiol as E2, and estriol as E3. Particularly, estradiol exists as 17α and 17β-estradiol, of which 17β-estradiol is the most prevalent and most potent female gonadal hormone, followed by estrone and estriol (33). Recently, it has been revealed that the role of estrogen is not limited to reproductive function. *Via* the bloodstream, estrogen is distributed to various tissues, such as the cardiovascular system, immune system, and CNS, where it exerts distinct effects (34, 35).

Estrogen exerts its functions by binding to estrogen receptors (ERs), which are present in several tissues, including the brain parenchyma (36). To date, three types of ERs have been identified: ERα, ERβ, and G-protein coupled receptors (37–39). Reportedly, estrogen mediates its effects *via* two signaling pathways (40). The first is the genomic pathway, which involves ERα and ERβ. After the binding of estrogen to ER, the activated receptor forms a homodimer and is delivered into the cell nucleus. Here, estrogen further binds to the ERE in the promoter site of various genes and serves as a transcription factor (41). The second way by which estrogen elicits its effects is through a non-genomic mechanism. In this mechanism, the ligand–ER dimer can locate itself in the cytoplasm or at the membrane, eventually activating the downstream protein kinases and phosphatases (42).

## 5 Regulatory role of 17β-estradiol in neuroinflammation

Ischemic stroke damages the ischemic core in the brain owing to the sudden deprivation of blood flow, oxygen, and nutrients (43, 44). A variety of ischemic cascade reactions can be observed within a few minutes after stroke onset, including increased oxidative stress and mitochondrial dysfunction, which eventually lead to cell apoptosis. These cascade reactions also include cell death-associated damage-associated molecular patterns (DAMP) and subsequent inflammatory responses (45). DAMPs promote the local activation of microglia and induce the recruitment of leukocytes (46). In this microenvironment, immune cells secrete pro-inflammatory cytokines, such as IL-1β, IL-6, and TNF-α, which induce local or systemic inflammatory responses (46, 47). Accumulative evidence has revealed that such immune responses can aggravate cerebral ischemic injuries as well as promote nerve regeneration, attenuate inflammation, and improve tissue repair after stroke (48, 49). In addition, the such inflammatory responses in brain can be observed in several conditions, such as infection, and are often associated with an increased risk of ischemic stroke. In line with this, a study has revealed that approximately 30% patients developed stroke during infection (50).

While the protective effects of 17β-estradiol after stroke are partially mediated by the neuroprotective pathways in the brain and immune regulation, brain injuries and tissue repairs are dependent on the immune responses after stroke onset. Specifically, the administration of 17β-estradiol after stroke can protect neural function and promote recovery through immune regulation (51). Reportedly, 17β-estradiol regulates inflammation *via* the activation of macrophages and release of anti-inflammatory cytokines, including IL-10 and TGF-β (52) These inflammatory responses observed in response to 17β-estradiol differ with sex throughout the lifespan and may contribute to the sexually dimorphic responses observed after stroke. However, there is still limited understanding regarding the relationship between sex hormones and immune regulation.

### 5.1 Estrogen and innate immunity

Stroke is associated with a significant increase in estrogen levels, which suggests an immediate physiological reaction following brain injury. The rapid, local production of estrogen has been reported to be associated with the activation of innate immunity (53). Reportedly, 17β-estradiol can inhibit the production and release of pro-inflammatory cytokines, including IL-1β, IL-6, and TNFα. This explains the role of 17β-estradiol in modulating immune responses, implicating its use as a therapeutic strategy (54).

#### 5.1.1 Estrogen and microglia

Microglia are derived from the primary myeloid precursors and are the resident immune cells in CNS (55). Accordingly, the critical role of microglia in brain injuries has been extensively explored. It has been reported that following brain injury, the number of microglia and the intensity of immune responses differ based on sex (56). A preclinical study has demonstrated that female mice have more microglia in the hippocampus than male mice. In addition, old female mice were found to have more microglia than younger ones, which accounts for the production and release of sex hormones (57). Moreover, according to an experimental study, the administration of estrogen can suppress microglial proliferation (58). In OVX female mice, ERT was found to reduce the number of microglial cells in the aged group. Furthermore, ovariectomy was found to increase the number of microglia and abrogate the restraining effects on microglial activation (59). Reportedly, estrogen induces the maintenance of resting phenotype with ramified morphology and downregulates MHCII expression (60). Furthermore, the ERs preset on microglia allow for estrogen to mediate its effects. In mouse models of middle cerebral artery occlusion, ERα knockout was found to activate the microglia and further induce larger infarcts. These additionally provide evidence for an ADIOL/ERβ/CtBP-transcription pathway that regulates inflammatory responses in microglia and can be targeted by selective ERβ modulators (61). Moreover, estrogen can inhibit microglial activation and reduce the level of proinflammatory cytokines, thus mediating neuroprotective effects through both ERα and ERβ activation (62–64).

In animal models of traumatic brain injury, estrogen was found to inhibit TLR4 and NF-κB protein expression; reduce the expression of the proinflammatory factors IL-1β, IL-6, and TNF-α; and decrease the number of complement C3d/GFAP-positive cells and complement C3d protein expression (65). Similarly, in animal models of spinal cord injury, microglial and astrocyte activation were both significantly inhibited, along with attenuation of the secretion of inflammatory mediators (66). Specifically, the location of ERβ in the microglial cytoplasm suggests the involvement of the nonclassical effects of estrogen on microglia (67). To this extent, a study has demonstrated that in microglial cells, estrogen-mediated anti-inflammatory pathways are mediated by the activation of the mitogen-activated protein kinase (MAPK) signaling pathway (68, 69). Notably, ERT has been found to stimulate early postischemic expression of bcl-2 and bfl-1 and reduce brain injury (70). In addition, estrogen can significantly downregulate factors mediating adaptive immunity in microglial cells; this highlights the multi-faceted regulatory effects of estrogen on microglial parameters related to antigen presentation and T-cell interaction (71) (**Table 1**).

**Table 1:** Anti-inflammatory effects of estrogen on microglia.

Year	Species	Treatment	Effects on inflammation	Reference
2000	Rat, mouse	17β-estradiol + LPS	Estrogen receptor-dependent activation of MAP kinase-mediated anti-inflammatory pathways in microglial cells.	(68)
2001	Rat, mouse	17β-estradiol + LPS	Pre-treatment of microglial cells with physiological concentrations of 17β-estradiol suppressed TAT-mediated microglial activation by interfering with TAT-induced MAPK activation.	(69)
2001	Rat	17β-estradiol + LPS	Inhibited the expression of iNOS, PGE2, and MMP-9.	(62)
2004	Rat	17β-estradiol + hypercholesteremia	Estrogen replacement stimulated early post-ischemic expression of *bcl-2* and *bfl-1* and reduced stroke-related damages.	(70)
2005	Rat	17β-estradiol + LPS	Estrogen inhibits microglial activation and thus exerts neuroprotective effects through both ERα and ERβ activation.	(67)
2005	Mouse	17β-estradiol + LPS	Estrogen can significantly decrease adaptive immunity in microglial cells, highlighting its multi-faceted regulatory effects ranging from microglial activation to antigen presentation and T-cell interaction.	(71)
2006	Mouse	17β-estradiol + LPS	Inhibited the expression of CCL2, MIP-2, and TNF-α.	(63)
2011	Mouse, human	5-androsten-3β, 17β-diol + LPS	Suppressed inflammatory responses and the microglial expression of IL-1β, IL-6, IL-23, and iNOS.	(61)
2016	Mouse	17β-estradiol	Reduced the level of proinflammatory cytokines, including IL-1β and TNF-α.	(64)
2018	Mouse	17β-estradiol	Significantly inhibited both microglial and astrocyte activation and attenuated the activity of inflammatory mediators.	(66)
2021	Mouse	17β-estradiol	Inhibited the protein level of TLR4 and NF-κB, reduced the expression of IL-1β, IL-6, and TNF-α, and decreased the number of complement C3d/GFAP-positive cells and the protein level of complement C3d.	(65)

LPS, Lipopolysaccharid; MAPK, Mitogen-activated protein kinase; TAT, Transactivator of transcription; iNOS, Inducible nitric oxide synthase; PGE2, Prostaglandin E2; MMP-9, Matrix metalloproteinase-9; BCL-2, B-cell lymphoma-2; ER, Estrogen receptor; CCL2, Chemokine ligand 2; MIP-2, Macrophage inflammatory protein 2; TNF-α, Tumor necrosis factor alpha; IL-1β, Interleukin 1 beta; NF-κB, Nuclear transcription factor kappa B.

#### 5.1.2 Estrogen and monocytes

Monocytes and macrophages play an important role in the resolution of inflammation by scavenging apoptotic neutrophils. In response to inflammatory signals from injury sites, monocytes are rapidly mobilized. Although it has been reported that monocyte infiltration in the injured brain mediates both beneficial and detrimental effects on immune regulation after stroke, the number of monocytes is positively associated with the risk of post-stroke infection (72). Reportedly, estrogen affects the functioning of monocytes by significantly altering myelopoiesis and monocyte migration. Specifically, during ovulation and gestation in females, the count of circulating monocytes is much higher than during other stages of the reproductive cycle. Estrogen also affects monocyte adhesion (73) by reducing the migratory and adhesive capacity of monocytes. This way estrogen limits stroke-induced inflammatory reactions, further alleviating the cerebral ischemia-reperfusion injury and selectively suppressing the activation of the neuroinflammatory cascade (74). In addition, several studies have demonstrated the anti-inflammatory effects of estrogen on monocytes. It has been reported that 17β-estradiol can induce the formation of vascular endothelial growth factor, while dihydrotestosterone can antagonize the effect of 17β-estradiol by regulating adenylate cyclase in THP-1 cells, which is mediated by GPR130 (75, 76). Another study demonstrated that a physiological dose of estrogen acutely stimulates nitric oxide release from human monocytes by activating estrogen surface receptors that are coupled to increases in intracellular calcium (77). Moreover, estrogen was found to inhibit proinflammatory cytokine release from activated monocytes partly by modulating CD16 expression (78, 79). In addition, at physiological concentrations, estradiol mediates monocyte adhesion as well as basal and hypercholesterolemia-induced increases in CXCR2 and MCP-1 expression (80–82) (**Table 2**). These data highlight that estrogen plays a role in post-stroke brain injury by significantly affecting monocytes and their functioning.

**Table 2:** Anti-inflammatory effects of estrogen on monocytes.

Year	Species	Treatment	Effects on inflammation	Reference
1998	Rabbit	17β-estradiol + hypercholesteremia	Basal and hypercholesterolemia-induced increases in MCP-1 protein are decreased by physiological concentrations of estradiol.	(80)
1999	Human	17β-estradiol	A physiological dose of estrogen acutely stimulated NO release from monocytes by activating an estrogen surface receptor.	(76)
1999	Rabbit	17β-estradiol	Inhibited monocyte adhesion by inhibiting VCAM-1 expression.	(81)
2002	Human	17β-estradiol	Increased NGF and vascular endothelial growth factor levels in THP1 cells	(75)
2003	Human	17β-estradiol	Increased NGF levels in THP1 cells	(76)
2003	Rat	17β-estradiol + hypercholesteremia	Physiological concentrations of estradiol modulated basal and hypercholesterolemia-induced increases in chemokine receptor CXCR2.	(82)
2004	Human	17β-estradiol	Decreased levels of TNFα, IL-1β, and IL-6 *via* the modulation of CD16 expression.	(78)
2007	Human	17β-estradiol + LPS	Decreased CXCL8 expression and inhibited LPS-activated monocytes.	(79)
2020	Rat	17β-estradiol	Alleviated cerebral ischemia-reperfusion injury and selectively suppressed the activation of the neuroinflammatory cascade.	(74)

MCP-1, Monocyte chemoattratctant protein-1; NO, Nitric oxide; VCAM-1, Vascular cell adhesion molecule 1; NGF, Nerve growth factor; CXCL, C-X-C motif chemokine ligand.

#### 5.1.3 Estrogen and macrophages

Hematopoietic stem cell-derived macrophages are another type of resident immune cells, apart from microglia, in CNS (83). These resident macrophages and microglia share overlapping features and have similar biomarkers, which complicate the assessment of their unique roles in estrogen-mediated neuroprotective effects. In addition, resident macrophages can continually renew from the bone marrow, unlike microglia (84, 85). So far, the potential role of resident macrophages is limited and based on the study of peripheral macrophages and cell lines. Therefore, accurate discrimination between the roles of microglia and macrophages after stroke requires further study. Reportedly, the proliferation and function of resident macrophages are associated with sex. A recent study focused on the changes in cell composition and immune function with sex and revealed higher resident leukocytes in the pleural and peritoneal cavities in females (86). In addition, resident macrophages had higher Toll-like receptor (TLR) expression and greater phagocytic and NADPH oxidase activities in females than in males (87). Reportedly, OVX can abrogate such effects caused by sex differences as well as regulate chemokine function, and macrophage count in females to the level observed in males. Interestingly, it has been revealed that OVX does not significantly alter T lymphocyte counts, suggesting no association of these lymphocytes with gonadal steroids (88). So far, although the role of sex differences in T lymphocyte populations has been explored in several autoimmune diseases, their effects in patients with stroke have not been fully investigated. Moreover, 17β-estradiol has been shown to inhibit the expression of inflammatory genes (TNF-α, IL-1β, MIP-2, and MCP-1) by controlling NF-κB or MAPK signaling pathway and attenuating H3 and H4 histone acetylation as well as cAMP response element binding protein-binding protein in macrophages (89–95) (**Table 3**). The above mentioned results of studies demonstrated the pivotal role of macrophages in inflammatory response post-ischemic stroke.

**Table 3:** Anti-inflammatory effects of estrogen on macrophages.

Year	Species	Treatment	Effects on inflammation	Reference
2004	Mouse	17β-estradiol + IFNγ	Attenuated H3 and H4 histone acetylation and cAMP response element binding protein to inhibit class II MHC expression.	(90)
2005	Mouse	17β-estradiol + LPS	Inhibited inflammatory gene expression by controlling NF-κB intracellular localization.	(89)
2006	Mouse	17β-estradiol	Elevated MyD88 and Src expression.	(94)
2007	Mouse, Rat	17β-estradiol	Mediated salutary effects on macrophage cytokine production *via* the normalization of MAPK signaling pathway.	(93)
2008	Mouse	17β-estradiol + H_2_O_2_	Inhibited cytokine production mediated *via* TNF-α, IL-1β, MIP-2, and MCP-1.	(91)
2010	Human	17β-estradiol + LPS	Repressed NF-κB activation through the induction of kappaB-Ras2.	(92)
2011	Mouse	Physiological estrogen	Increased the expression of TLR2/3/4, Myd88, CCL2, CX3CR1, CXCL1, CXCL12, CCR1, CCR2, CXCR4, and NAPDH oxidase.	(86)
2022	Mouse	17β-estradiol	Suppressed neutrophil- and macrophage-mediated production of IL-1β.	(95)

MHC, Major histocompatibility complex; cAMP, Cyclic adenosine monophosphate; TLR, Toll-like receptors; NADPH, Nicotinamide adenine dinucleotide phosphate oxidase.

#### 5.1.4 Estrogen and neutrophils

Stroke is associated with the disruption of blood–brain barrier, following which neutrophils immediately migrate into the injured sites (96). At the early stage, neutrophil infiltration can activate monocyte-derived macrophages to scavenge debris. Moreover, at sites of inflammation, neutrophils release pro-inflammatory cytokines that recruit more leukocytes. However, excessive neutrophil infiltration exacerbates brain injury (97). According to clinical studies, neutrophil accumulation is related to poor prognosis in patients with brain injuries (98). Several studies have revealed a positive correlation between estrogen levels and neutrophil counts (95, 99). To this extent, a study has reported that in females, myeloperoxidase activity in neutrophils is higher than that in males. Moreover, estrogen has been reported to promote the degranulation and release of myeloperoxidase, elastase, cytokine-induced neutrophil chemo-attractant-1 (CINC)-1, CINC-3, and intercellular adhesion molecule-1 as well as increase nNOS expression and superoxide levels in neutrophils (100–103). The dynamics of neutrophil infiltration after stroke into the brain parenchyma and subsequent neutrophil-mediated responses have yet to be completely determined (104). Available studies suggest that 17β-estradiol can inhibit the synthesis of neutrophil chemoattractants at ischemic sites and regulate excessive neutrophil infiltration. Moreover, 17β-estradiol can inhibit neutrophil adhesion to endothelial cells; mediate the clearance of neutrophils; and inhibit the expression of chemotaxis, IL-1, IL-6, and CINC-2α to neutrophils by binding to Erα. It can additionally inhibit IFN-γ-induced Stat1 phosphorylation as well as downregulate CD40 and CD40L protein expression (105–107) (**Table 4**).

**Table 4:** Anti-inflammatory effects of estrogen on neutrophils.

Year	Species	Treatment	Effects on inflammation	Reference
1999	Human	17β-estradiol	Increased nNOS and decreased CD18 expression.	(102)
1999	Human	Physiological estrogen	Promoted MPO activity	(103)
2004	Human	17β-estradiol + fMLP	Promoted MPO activity, elastase levels, superoxide levels, and LDL oxidation.	(100)
2004	Rat	17β-estradiol	Decreased chemotaxis and levels of IL-1, IL-6, and CINC-2α in neutrophils.	(106)
2006	Rat	17β-estradiol	Promoted MPO activity and CINC-1, CINC-3, and ICAM-1 expression.	(101)
2006	Pig	17β-estradiol + IFNγ	17β-estradiol binding to ERα blocked IFN-γ-induced Stat1 phosphorylation, inhibited CD40 and CD40L protein expression, and prevented neutrophil adhesion onto ECs.	(105)
2011	Human	17β-estradiol	Increased nNOS annexin A1 expression and inhibited neutrophil adhesion onto ECs.	(107)

MPO, Myeloperoxidase; CINC, Cytokine-induced neutrophil chemoattractant; EC, Endothelial cell; nNOS, Nerve nitric oxide synthetase; IFN-γ, Interferon gamma.

#### 5.1.5 Estrogen and dendritic cells

DCs are considered the only antigen-presenting cells that are involved in post-stroke injury and have a remarkable ability to activate memory and naïve T lymphocytes (108). DCs are a member of innate immunity and play a critical role in the phagocytosis and release of inflammatory factors. They additionally play a unique role in bridging innate and adaptive immunity (109), owing to which these cells have been correlated in a wide range of diseases, including atherosclerosis, various cancers, and more recently, stroke (110). Accumulative evidence has revealed increased recruitment of DCs and their potential role in brain injury following stroke onset. Moreover, an animal study has revealed the presence of DCs with high MHCII and CD80 expression at 72 hours after reperfusion and has demonstrated their association with lymphocyte migration in a time-dependent manner (111). In general, increased DC infiltration in the brain parenchyma after stroke may be positively correlated to the degree of injury. Estrogen administration in experimental autoimmune encephalomyelitis (EAE) mice has demonstrated significant improvement in their neurobehavior and outcomes as well as a marked reduction in the DC count. In addition, 17β-estradiol was found to upregulate the expression of MHCII, IL-10, CD40, CD83, CD54, IL-6, IL-8, MCP-1, and osteoprotegerin; upregulate stimulative capacity, migratory activity, and antigen-presenting capacity; and inhibit cell apoptosis (112–115). DCs are considered the link between innate and adaptive immunity. Moreover, DCs harbor antigens specific to T lymphocytes, which on activation initiate adaptive immune responses. Correspondingly, estrogen can induce DC differentiation and MHC expression, which facilitate T lymphocyte-mediated immune responses in the brain. Specifically, 17β-estradiol treatment is of great clinical value in regulating DCs. A study has reported that 17β-estradiol administration induces both the TLRs for IL-23 production in OX62+ DCs, thus stimulating IL-6 and IL-1β production (116). In addition, 17β-estradiol induces CD86 expression in CD103^+^ DCs after allergen-mediated upregulation of IL-5 in CD4^+^ T cells (117) (**Table 5**).

**Table 5:** Anti-inflammatory effects of estrogen on dendritic cells and adaptive immunity.

Year	Species	Treatment	Effects on inflammation	Reference
2004	Human	17β-estradiol	Increased IL-6, IL-8, MCP-1, and osteoprotegerin levels as well as promoted stimulative capacity and migratory activity.	(112)
2005	Mouse	17β-estradiol	Increased MHCII, IL-6, IL-10, CD40, and CD54 levels; viability; and stimulative capacity.	(113)
2006	Mouse	17β-estradiol	Increased MHCII, IL-6, IL-10, CD40, and CD54 levels; viability; and stimulative capacity.	(114)
2006	Mouse	17β-estradiol + LPS	Upregulated PD-1 expression and Treg activity and inhibited T lymphocyte proliferation.	(118)
2008	Mouse	17β-estradiol + LPS	Increased MHCII, CD83, CD40, TNF, IL-6, and IL-12p40 expression; promoted antigen-presenting capacity; and inhibited cell apoptosis.	(115)
2011	Mouse	17β-estradiol	Promoted Th2 response and increased CD80, CD86, PD-L1/2, B7-H3/4, IL-10, and TGF-β expression.	(119)
2016	Rat	17β-estradiol	Promoted the stimulatory action of both TLRs for IL-23 production in OX62+ DCs as well as augmented their stimulatory effects on IL-6 and IL-1β production.	(116)
2018	Mouse	17β-estradiol	Enhanced CD86 expression in CD103^+^ DCs and upregulated IL-5 production in CD4^+^ T cells.	(117)

### 5.2 Estrogen and adaptive immunity

Evidence shows that 17β-estradiol can modulate the immune microenvironment and control the infiltration of T lymphocytes in the brain. The infiltration of cytotoxic T lymphocytes (CD8+) exacerbates brain injury, while T regulatory cells (Tregs) mediate anti-inflammatory effects and are responsible for reducing lesion volume and improving prognosis after stroke (120). 17β-estradiol administration can promote Treg responses through the inhibition of Th1- and Th17-derived cytokines or by directly promoting the proliferation of Tregs, upregulating PD-1 expression, and downregulating T lymphocyte proliferation (118, 121). Additionally, 17β-estradiol can promote Th2 response, thus upregulating the expression of CD80, CD86, PD-L1/2, B7-H3/4, IL-10, and TGF-β (119). Estrogen also plays a critical role in the regulation of B lymphocytes. Previous studies have shown that estradiol treatment protected purified B cells from apoptosis. Similarly, estradiol was shown to protect mice splenic B cells of from serum-deficiency-induced apoptosis; however, no effect was observed on the proliferation of B cells (122).

## 6 ERT: estrogen and immune regulation

It is well known that ERα is widely localized in the brain, including the forebrain, hypothalamus, and hippocampus (123). The binding of estrogen to ERα in the brain is responsible for several effects in both the physiological and pathological states. Reportedly, several genes are associated with neuronal survival and are regulated by an ERE-containing promoter. In particular, 17β-estradiol can promote the transcription of such genes and exert neuroprotective roles. This has been demonstrated in a study that found that 17β-estradiol could promote the transcription of a wide range of genes (124). After brain injury, 17β-estradiol administration can promote the upregulation of cell survival proteins, including phosphoinositide 3-kinase, cyclic-AMP response element-binding protein (CREB), Bcl-2, Bcl-x, c-fos, and c-jun. Moreover, 17β-estradiol inhibits the expression of apoptosis-related proteins, including Fas, FADD, and Bax, thus subsequently downregulating Cyto C release (125). Accumulative evidence has revealed the effects of downstream signaling of 17β-estradiol. Administration of 17β-estradiol can activate the MAPK pathway and promote the phosphorylation of CREB (126). In addition, it can inhibit the activation of caspase-3/8 and thus suppress ischemia-induced acetylation of p53 (127).

In the context of stroke, the neuroprotective effects of 17β-estradiol have been demonstrated in OVX animal models of ischemic stroke. OVX animals are the widely accepted model for post-menopausal women, as the removal of ovaries in female animals effectively mimics the diminished estrogen levels observed in post-menopausal women (128). Based on an extensive review of experimental studies, estrogen was found to reduce lesion volume after transient or permanent cerebral ischemic stroke in a dose-dependent manner. Therefore, pre-treatment with estrogen is considered as a prevention strategy for stroke onset, while post-treatment with estrogen is a potential therapeutic strategy (129).

Currently, the positive effects of estrogen in patients with stroke have been well investigated. However, according to the results of the Women’s Estrogen for Stroke Trial and the Women’s Health Initiative trial, estrogen may increase the incident risk of stroke, which limits the establishment of a therapeutic strategy for stroke based on estrogen (130). Of note, the therapeutic time window and dose are the critical factors in the treatment of patients with stroke. A previous clinical trial has reported that early administration of ERT was associated with a lower risk of stroke onset than later administration in the post-menopause phase (131). Moreover, lower doses of estrogen exert stronger neuroprotective effects against stroke.

Long-term administration of high-dose, micronized estradiol in healthy women remarkedly increases the level of C-reactive protein (CRP), a bio-marker used to assess vascular risk (132). Similarly, the levels of endogenous estradiol are negatively associated with CRP levels in young women, suggesting the anti-inflammatory effects of estrogen (133). It has been suggested that ERT should be started immediately at menopause to achieve maximal beneficial effect.

## 7 Potential therapeutic effects of estrogen in patients with stroke

According to previous studies, 17β-estradiol can effectively attenuate brain injuries, reduce infarct area, and promote recovery in animal stroke models. However, these positive effects have not been well supported by clinical data (134). Furthermore, a variety of clinical trials have reported that ERT does not induce protective effects against primary or secondary brain injuries and instead increases the risk of stroke onset (130, 135).

The difference in the effectiveness of estrogen observed in experimental and clinical studies may be attributable to differences in conditions being treated. Experimental studies assessed the treatment of ischemic cerebral injury, whereas the clinical trials focused on the prevention of stroke, which has not been thoroughly explored in experimental studies. In clinical trials, time, duration, and dosage are the important factors that mediate the negative effects of ERT (136). Based on clinical data, ERT can increase the risk of venous thrombosis, whereas a percutaneous approach to estrogen administration can reduce this risk (137, 138). Therefore, low dosage, short-term treatment, and a percutaneous approach will avoid increasing the risk of stroke onset and may formulate a safe alternative for the prevention and treatment of stroke. In a nested case-control study, short-term percutaneous treatment with estrogen was considered the safest choice against ischemic stroke (139). Compared with the oral approach, the percutaneous approach avoids the first-pass metabolism of estrogen in the liver. This prevents interaction with coagulation factors, inflammatory cytokines, and sex hormone binding protein, which reduces the risk of venous thromboembolism, which is often observed with oral estrogen. According to a clinical study, estrogen levels increase after stroke. Moreover, inflammatory control and the addition of exogenous hormones are likely to improve neural function in elderly male patients with stroke (140).

## 8 Conclusion

Generally, the host response to sterile inflammation is considered as a beneficial reaction; however, a hyper-immune response or altered signaling can lead to homeostatic imbalance and induce further chronic inflammation. Evidence has revealed that prolonged neuroinflammation is detrimental to clinical prognosis of stroke. Moreover, stroke-induced immunosuppression can increase susceptibility to infections, which complicates treatment. Because of the delayed diagnosis and subsequent delay in treatment, it is essential to establish new treatments with wider treatment windows. Neuro-inflammation is an attractive target for treatment of stroke owing to its wide therapeutic window. Therefore, several studies have attempted to identify novel neuroprotective strategies to target immune reactions in patients with stroke.

Previous experimental studies have demonstrated that estrogen is a potent immunomodulator and is considered a neuro-protective molecule in ischemic stroke (34, 35). Specifically, after stroke onset, the release of pro-inflammatory cytokines can aggravate brain damage. Although the use of estrogen or its analogs to regulate immune responses requires further exploration, ERT could be a potential treatment that targets immune responses in patients with stroke. Irrespective, experimental and clinical studies have not reached a consensus regarding the role of estrogen in alleviating post-stroke brain injury. This may be attributed to the conditions being targeted, as experimental research is mainly aimed at the treatment of acute ischemic stroke, while clinical trials are aimed at the prevention of primary or secondary stroke (141, 142). Furthermore, 17β-estradiol can regulate the immune system by inhibiting the release of pro-inflammatory cytokines and attenuating inflammatory reactions (143, 144). Despite the limited pre-clinical data regarding the effects of 17β-estradiol on immune regulation, the positive effects of 17β-estradiol on attenuating inflammatory reactions have been proven. Understanding the effects of 17β-estradiol provides the opportunity to explore novel therapeutic strategies while avoiding the controversial off-target effects of estrogen. Irrespective, it is necessary to establish more animal models to replicate the clinical conditions to provide a basis for future clinical trials.

## Author contributions

All authors contributed to the general design of the study. LX conceptualized the review. XZ drafted the review. YL prepared the tables. YS, YL and LX revised the manuscript. All authors contributed to the article and approved the submitted version.
